# The stigma of underperformance in assessment and remediation

**DOI:** 10.1007/s10459-024-10382-8

**Published:** 2024-10-16

**Authors:** Damian J. Castanelli, Elizabeth Molloy, Margaret Bearman

**Affiliations:** 1 School of Clinical Sciences at Monash Health, Monash University, Clayton, VIC Australia; 2 Department of Anaesthesia & Perioperative Medicine, Monash Health, Clayton, VIC Australia; 3 Department of Medical Education, Melbourne Medical School, University of Melbourne, Melbourne, VIC Australia; 4 Centre for Research & Assessment in Digital Learning (CRADLE), Deakin University, Geelong, VIC Australia

**Keywords:** Stigma, Remediation, Failure to fail, Competence, Post-graduate medical education

## Abstract

The stigma of underperformance is widely acknowledged but seldom explored. ‘Failure to fail’ is a perennial problem in health professions education, and learner remediation continues to tax supervisors. In this study, we draw on Goffman’s seminal work on stigma to explore supervisors’ accounts of judging performance and managing remediation in specialty anesthesia training in Australia and New Zealand. In doing so, we focus on what Goffman calls a “stigma theory” to explain the supervisors’ reported practices. We performed a secondary analysis of nineteen interviews originally gathered using purposive sampling to explore how assessment decisions were made. We conducted a theory-informed thematic analysis of the supervisors’ accounts to identify signifiers of stigma and underlying structures and beliefs. From both deductive and inductive analysis, we developed themes that demonstrate how the stigma of underperformance influences and is induced by supervisors’ reticence to discuss underperformance, their desire to conceal remediation, and their differential treatment of trainees. We also found that accounts of trainees ‘lacking insight’ resembled stigma-induced stereotyping. We argue from our data that our cultural expectations of perfectionism propagate a stigma that undermines our efforts to remediate underperformance and that our remediation practices inadvertently induce stigma. We suggest that a multifaceted approach using both individual and collective action is necessary to change both culture and practice and encourage the normalisation of remediation.

## Introduction

Supervisors in clinical practice spend considerable time and energy considering– and avoiding– the ‘problem’ of underperformance (Bearman et al., 2013; Yepes-Rios et al., 2016). However, as they recognise failure, they can become angry, using “harsh, unequivocal language” (Gingerich et al., 2022, p5). As Gingerich et al. describe it, supervisors reach a tipping point where they switch from being ambivalent to using stigmatising language (e.g. “he’s a disaster”, “I probably would have opened a vein if I had to supervise him for six months”). Stigma associated with serious underperformance has been recognised as an impediment to helping trainees in remediation (Chou et al., 2019; Price et al., 2021). Indeed, stigma influences outcomes for stigmatised individuals in fields outside education, such as healthcare (Addison, 2023). We suggest stigmatising language and behaviours, in particular avoidance, have the potential to result in incompetent practitioners or traumatised trainees.

This paper explores how the concept of stigma helps us understand how supervisors are judging and remediating underperformance, in the context of postgraduate anaesthesia training. Fundamental to such training programs is deciding whether a trainee’s performance is satisfactory so that they can progress to the next stage of learning. As stigma is, at heart, a sociocultural phenomenon, we take a sociocultural perspective whereby learning can be conceptualised as a trajectory into a community of practitioners (Lave & Wenger, 1991). Trainees’ paths from novice to full participant are neither uniform nor linear. Trainees have their own histories and circumstances, and their responses to the affordances of their context dictate their learning trajectory (Billett, 2009). Yet not every path leads to the desired destination; educators need to delineate when a trainee’s individual path deviates from the ‘broad corridors of acceptability’ (Ellaway, 2023, p4) and is at risk of failure. Either the performance issues are resolved and the trainee can return to ‘normal practice’, or the deficiencies in their work are judged irremediable and they cannot continue training (Ellaway, 2023). The literature repeatedly emphasises the aspirational need to normalise remediation (Kalet et al., 2017; Kuehl & Gisondi, 2015) but this is difficult to do in practice (Mills, 2023).

Stigma has been a prominent concept in social sciences since Goffman highlighted it in 1963 (Goffman, 1963). Stigma is a social construct, describing processes that occur in a sociocultural environment, where a perceived difference results in the labelling and stereotyping of an individual. This stigmatising difference is ‘incongruous with our stereotype of what a given type of individual should be’ (Goffman, 1963, p2). The processes provoked by stigma promote discrimination, threaten social identity and may produce status loss and, ultimately, social exclusion (Goffman, 1963; Link & Phelan, 2001; Yang et al., 2007).

The impact of stigma is mediated by whether the signifying difference is known or unknown. Goffman noted that those with noticeable or disclosed differences are ‘discredited’ (e.g. physical disability), while those with concealed or undisclosed differences are ‘discreditable’ (e.g. mental health disability) (Goffman, 1963). Where the stigmatising difference is not visible, control of the information and its potential disclosure, what Goffman termed its ‘known-about-ness’, becomes important (Goffman, 1963, p49).

The stigmatising difference disrupts our underlying belief or expectation. We respond by turning away or separating ourselves from the stigmatised person, often invoking a ‘stigma theory’ of their lesser status to explain our actions (Goffman, 1963). The same processes act upon those stigmatised, who may internalise prejudiced views or interpret others’ normal actions as stigmatising in a concept known as self-stigma (Goffman, 1963; Krug et al., 2019). While individuals enact it, stigma stems from underlying beliefs, policies and structures (Charmaz, 2019). Hence, stigma has both a relational/interpersonal element and a cultural or structural element. Combatting stigma is challenging since it requires recognising and changing entrenched attitudes and beliefs (Phelan et al., 2014). Clarifying how the stigma of underperformance arises and the role it plays in remediation may help us combat stigma and normalise the performance improvement process.

In this paper, we aim to illuminate how supervisors’ behaviour toward ‘underperforming trainees’ (note, judged as) reflects or induces stigma. Our research question is: How do stigmatising actions, including language, feature in supervisors’ descriptions of how they work with underperformance?

## Methods

The context for this study is postgraduate anaesthesia education in Australia and New Zealand, which is governed by a binational college. We conducted a secondary analysis of interview data to produce this paper. The interviewees were Supervisors of Training recruited using purposive maximal variation sampling (Patton, 2015), as previously described (Castanelli et al., 2019, 2020). Supervisors of Training, referred to hereafter as ‘Supervisors’, are specialist anaesthetists responsible for training within their department. They oversee each trainee’s clinical performance, perform regular clinical placement reviews, judge whether trainee progress is satisfactory, and manage remediation when required. Supervisors are individually responsible for summative assessment decisions in the workplace, as there are no competence committees.

This study received ethics approval from Monash University (Reference: 2016000919) and the University of Auckland (Reference: 017408).

We conceived the research project of which this study forms a part from a particular worldview; in the social sphere, individuals create their own interpretation of reality through interaction with others in a community with shared understandings (Crotty, 1998; Tavakol & Sandars, 2014). We recognise that our interview data reflects our participants’ socially constructed meaning taken from their experiences rather than any ‘true’ record of what occurred. We do not suggest that supervisors are of poor quality or that their actions are uncaring; instead, we seek to understand how a broader culture of shared beliefs and practices plays out in supervision.

There were nineteen interviews that explored how supervisors understood their role and made progress decisions in practice. In the original analyses, we used the methods of thematic analysis described by Braun and Clark (Braun & Clarke, 2006; Braun et al., 2015). Through these inductive analyses, the results of which have been previously published (Castanelli et al., 2019, 2020), we gained familiarity with the data and were alerted to the potential role of stigma in managing underperformance.

We subsequently returned to the data using theory-directed thematic analysis (Braun & Clarke, 2022). In this application of thematic analysis, the aim is to explore ‘how a theory or concept is evidenced’ (Braun & Clarke, 2022, p267) in the data. We started coding deductively, guided by the characteristics of stigma described above (Braun et al., 2015; Goffman, 1963). In particular, we looked at how ‘labelling’, ‘stereotyping’, ‘separation’, and ‘status loss’ with respect to underperformance appeared in our supervisors’ accounts (Link & Phelan, 2001) and noted stories of trainee actions that could suggest a response to stigma. We also attended to comments that reflected supervisors’ beliefs and any cultural or structural factors that might support stigma (Charmaz, 2019). We supplemented this deductive coding with inductive coding to capture nuances in the data that fell within and between these codes. Coding was facilitated using NVivo software (version 12, QSR International Pty Ltd, Melbourne, Vic., Australia). After coding was complete, DC performed a provisional analysis, generating provisional themes and a thematic structure from the codes, which was subsequently reviewed by the research team (Braun & Clarke, 2022). After further refinement during this review, the thematic structure and themes were finalised.

As researchers, we bring our own personal perspectives to our analysis. One author (DC) is a practicing anaesthesiologist and a supervisor like our participants. He thus provided insights based on his own experience and his role is why we studied this system. MB and EM have backgrounds in computer science and physiotherapy, respectively, and all three authors are PhD-qualified researchers with extensive experience in health professions education research and qualitative research methods.

The process of theory-directed thematic analysis required more reflexivity than other research we have performed together. We had multiple concerns reconciling our analytic and interpretative choices. We worried we might be restricting our interpretations, ‘forcing’ our data to fit a pre-conceived and partial story owing to the deductive nature of the early phase of analysis. Such an approach is inconsistent with our interpretivist research framing. Instead, we sought to remain open and curious, acknowledging that many factors are at play in the practices we are studying. We reminded ourselves that there are many ways in which data can be interpreted and that any account of a phenomenon will always be partial. We emphasised particular analytical moves. When looking at the data, we looked for exceptions; when we interpreted participants’ words and found that the meaning aligned with Goffman, we considered the counter-case. We also reminded ourselves, unless specifically articulated by participants, that we could not be sure *why* they took a particular approach. That is to say, supervisors may have undertaken stigmatising actions, but these might not be due to the perception of a stigmatising difference.

## Findings

We have framed our analysis around Goffman’s conception of stigma, and thus, we present our findings in relation to his theorising. Our first theme, *Spoiling*, presents the beliefs and expectations underlying the stigma of underperformance. Our subsequent themes reflect the way stigmatising language and actions permeated our participants’ stories of making performance judgements and managing remediation. In *Avoiding*, there was a reluctance to discuss underperformance, which might reflect or emphasise its discrediting nature. In *Concealing*, there was a need for secrecy and limiting disclosure. This included a reluctance to create official records that could hamper remedial interventions and antagonise other trainees. We noted remedial efforts led to *Stereotyping*, our fourth theme, signalling potentially more severe consequences such as the labelling of the trainee as ‘lacking insight’. Finally, labelling the trainee as underperforming resulted in differential treatment, or *Treating differently*. This special treatment manifested as more direction, more oversight, and re-allocation of work (most commonly restricting access to important tasks for working and development) that provoked a separation of the trainee from other department members.

### Spoiling

An ‘incongruous difference’ disrupting our ‘normative expectations’ (Goffman, 1963, p2) produces stigma. In other words, stigma is what Goffman calls a ‘spoiled identity’. If underperformance is stigmatising, then what identities does it disrupt? We found two underlying beliefs that might be disrupted by underperformance.

Firstly, underperformance may challenge the prevailing culture of perfectionism in healthcare. When discussing the deficiencies of workplace-based assessments, supervisors noted the difficulty they faced persuading specialists and trainees to embrace assessment for learning and to use the lower range of the entrustment scale. A quote from another supervisor explains why:

“Because most trainees are high achievers and so they are used to getting consistently high marks and even if it’s appropriate for them with that case to have a lower mark, then the trainees struggle with that and therefore the supervisors struggle to give it because they, you know, they feel that that’s not the way it should be.” (#10).

Supervisors faced similar difficulties themselves when discussing end of term assessments. The ‘way it should be’ extended to the performance feedback they gave, leading one to explain:

“If you don’t say very positive things, naturally the highly-strung trainees that we have get very concerned about that.” (#4).

Secondly, underperformance may challenge the expectation that trainees will progress smoothly through training in the minimal allowed time. This underlying belief was apparent when one supervisor discussed how they made progress decisions:

“The default’s a yes, because a vast majority of our trainees are good trainees and good people and good doctors, and I don’t expect to fail them. So, giving someone a fail is a quite big deal.” (#17).

Supervisors’ expectation that trainees would progress at the earliest opportunity was also evident when they wished to indicate what level the trainee had attained in the training program. Rather than refer to the trainee’s current stage of training, they routinely used the trainee’s year of training, e.g. “first year” or “fourth year”. When discussing the consequences of a trainee not being ready to progress, supervisors described how the trainee would “go into Extended Training” (#9). This is because the training program allowed trainees double the minimum time to complete the requirements of each training stage without penalty or requiring permission. However, training time beyond the minimum is officially labelled as ‘Extended Training’.

These underlying beliefs of trainee perfectionism and smooth progression in the minimum time appear to be the normative expectations that underperformance disrupts to initiate stigma.

### Avoiding

Our participants’ reports reflected a reluctance from trainees, supervisors, and their colleagues to discuss underperformance. Until the diagnosis of underperformance is communicated, the trainee inhabits the world of what Goffman calls ‘normals’; however, once diagnosed as underperforming, they are henceforth labelled with what might be referred to as a ‘deeply discrediting’ attribute (Goffman, 1963). Such avoidant behaviour is consistent with actions suggesting stigma, an avoidance of the stigmatising state.

We noted supervisors generally associated avoidant behaviours with underperformance. Whether this was a response to a stigmatising difference or avoidance for other reasons was unclear. Either way, it seemed to signal that underperformance was a ‘discrediting’ difference. For example, supervisors noted that some trainees avoided drawing attention to themselves, and their assumption was that this was a matter of underperformance. For example, if a trainee chose “doves” (#16) as workplace-based assessors or steered away from assessable tasks altogether:

“If you have a trainee who has only done one workplace-based assessment the whole term… that’s usually an indication that they are not doing well.” (#8).

Avoiding discussion of underperformance was also repeatedly noted: supervisors reported that their specialist colleagues were reticent to discuss underperformance with the trainee or record their views in writing, for example:

“Consultants don’t want to fail any of the trainees. They don’t want to have those difficult conversations.” (#10).

Whether or not the trainee was worthy of failure is immaterial here, the reluctance to have the “difficult conversation” could signal the ‘discrediting’ difference of underperformance. This reluctance is reinforced by supervisors’ description of delivering the diagnosis of underperformance as a “dreadful process” (#10):

“I find it really difficult. I wasn’t looking forward to it… I make sure I have tissues… and be empathic towards them… so that they don’t feel like it’s just a punishment session.” (#14).

The trauma for the trainee implied in supervisor accounts of relaying the underperformance diagnosis parallels what Goffman calls the “painfulness… of sudden stigmatization” that he says comes from the trainee’s knowledge of what follows (Goffman, 1963, p 132)– the “punishment” referred to by the supervisor. Indeed, the reluctance of supervisors to make and communicate the diagnosis, and of trainees and supervisors to help in detection, may reflect the general stigma attached to underperformance, or equally establish the discrediting nature of this difference and, in so doing, create stigma.

Fear of being labelled with a stigmatising difference leads those with other stigmatising attributes to avoid disclosure and fail to access help (Young et al., 2019), just as supervisors report underperforming trainees do. Applying stigma theory, labelling may expose the trainee to the “shared cultural beliefs” the community holds about underperformance and create a separation between them and the rest of the community, risking diminished status and social isolation (Phelan et al., 2014).

In subsequent themes, we will see whether such fears are reflected in our supervisors’ accounts.

### Concealing

If underperformance is stigmatising, it represents an invisible difference that, according to Goffman, generates the dilemma of disclosure. As he says, the stigmatised person is faced with the dilemma: ‘to tell or not to tell; to let on or not to let on; to lie or not to lie; and in each case, to whom, how, when, and where’ (Goffman, 1963, p42). As long as the signifying difference remains undisclosed, the person can avoid some of the impact of any attendant stigma. We saw in our data that concealing or managing the disclosure of underperformance was an important aspect of supervisors’ remediation practice.

Supervisors reported that they prioritised minimising disclosure in remediation:

“We maintained that anonymity and we pushed that this was a fair and private, obviously confidential process.” (#16).

Nevertheless, absolute secrecy was impossible if the trainee was to be supported in remediation; instead, knowledge of the trainee’s predicament was kept to as small a group as possible:

“One of the big challenges of keeping confidentiality where– but yeah, keeping things safe. So only those who need to know, know.” (#10).

The supervisors, in effect, contribute to forming what Goffman called a ‘protective circle’(Goffman, 1963, p97) of those who know and on whom the stigmatised individual can rely. One supervisor acknowledged that secrecy could make it challenging to explain treating the trainee differently to other trainees. Similarly, other anaesthetists supervising the trainee outside the protective circle might be left unaware, which precluded any extra support but avoided the concern that labelling the trainee as underperforming could prejudice future interactions:

“Asking for extra feedback and supervision, people, you know– putting someone in the spotlight can adversely affect their performance… because I don’t want for it to be a foregone conclusion, you know. I think if someone is flagged as being, you know, someone who’s not progressing or having trouble then often that can colour the feedback that you get.” (#10).

Thus, we see that concealing acts as a stigmatising behaviour. This belief is consistent with what we know of stigmatising conditions, where stigma leads to lower expectations of competence and lower performance judgements (Phelan et al., 2014). As outlined by this supervisor, these lower expectations may also be unconsciously shared by the stigmatised person, impacting their performance and contributing to poorer performance judgements.

The effort to conceal was considerable: some supervisors described an initial stage of remediation where they kept their own private notes. One noted: if “you type it into their record, it will alarm the trainee, plus alarm the next Supervisor of Training” (#4). Thus, in successful remediation, the formal training record might hold a “sanitised version of events” (#1), or no record at all.

The way supervisors manage the knowledge the trainee has underperformed is consistent with what Goffman describes as the discreditable person’s ‘masquerade’, where he handles ‘his risk by dividing the world into a large group to whom he tells nothing, and a small group to whom he tells all’ (Goffman, 1963, p95). Whether supervisors conceal and disclose underperformance in response to the stigma attached to underperformance or not, their actions contribute to the perception that underperformance is a difference that ought to be hidden and not discussed and thus contribute to the perception of stigma.

### Stereotyping

With labelling, the beliefs associated with the stigma become attached to the person, a process Goffman described as stereotyping (Goffman, 1963). Others therefore believe they already know what problems the stigmatised person faces and how they should face them: ‘To his other troubles, he must add that of being simultaneously pushed in several directions by professionals who tell him what he should do and feel about what he is and isn’t, and all this purportedly in his own interests’ (Goffman, 1963, p124). In our participants’ reported conversations with trainees planning remediation, supervisors afforded the trainee little opportunity to explain their perceived underperformance or help plan their remediation. Supervisors had already decided both the problem and how to best address it before meeting with the trainee. For example, one supervisor told how they organised to meet the trainee only after assuring themselves:

“Do I have a solution for this, you know? And will this solution, can it be implemented by this trainee?” (#18).

Rather than explore the trainee’s view, supervisors wanted the trainee to accept their narrative of the trainee’s performance ‘state’ and what steps the trainee should take to address it. One supervisor summarised this succinctly, telling the trainee:

“This is what we perceive as your issues, and this is what you can do to get better with them.” (#17).

‘Lack of insight’ provides a particular example of stereotyping common in underperformance (Bearman et al., 2018). From our interviews, supervisors equate trainee insight with accepting the supervisor’s view of their underperformance, how it has come about, and what should be done about it. Being judged to have insight, and hence conforming to supervisors’ views on how to behave, augured well for successful remediation:

“It always makes my job easier when they have insight, and even if they get very, you know, quite sentimental about it and quite teary, if they have got insight, I’m always pretty sure that they will settle down.” (#18).

In some supervisor reports, insight was something the trainee either possessed or didn’t, while in others it could be instilled if the supervisor could convince the trainee:

“I need to have all the evidence in place so that that the trainee can be provided with insight… a lot of it is having proof that there is a problem and having a process and a solution available.” (#4).

In one case, a supervisor reported that a trainee who initially lacked insight subsequently gained it:

“He started interrupting me… there was a lot of things that he said that I had clearly stated and [he] pretended to feign ignorance… [sometime later] that trainee said, “I guess I do have a problem because I feel that every six months or so, I get pulled into [a supervisor’s] office”, so… that message got through.” (#16).

Supervisors agreed that trainees who lacked insight were the most difficult to remediate. If the trainee was labelled as lacking insight, it implied that they did not accept they were underperforming or cooperate with the prescribed remediation. One supervisor provided a detailed example:

“Some issues were brought to the trainee’s attention, and after that it was very, very difficult to get the trainee to engage… it culminated in… an avalanche of feedback about different professional and clinical concerns, and… quite a concerted effort as [a supervisor] to engage early and to provide support and assistance… but a complete lack of insight and engagement from the trainee, an act of dishonesty and manipulation… a very difficult situation to deal with somebody who doesn’t want to engage in any processes that are designed to help them, and - and actively avoids them… it was incredibly stressful, because the trainee genuinely had no insight and continues to have absolutely no insight and coupled with that was very dishonest.” (#13).

Our supervisors’ views on those lacking insight concur with Goffman’s view of the stigmatised who ‘fail to adhere to the code’ (Goffman, 1963, p111). By failing to accept the supervisor’s view and advice on how to behave, trainees open themselves to being seen as ‘an impaired person, rigid, defensive, with inadequate inner resources’ (Goffman, 1963, p115). One supervisor used words such as “angry”, “defensive”, and “aggressive” (#15) in describing a trainee who they judged lacked insight. Unfortunately, in this case, a persistent lack of insight, from the supervisor’s viewpoint, resulted in unsuccessful remediation:

“Well, he subsequently unravelled actually… He never got back on to night shift and still continued to behave in the ways that he did… and he eventually ended up going through a disciplinary process… we were looking to exit him from training, but he actually just quit.” (#15).

The trainee’s unravelling and inability to change to meet the supervisor’s expectations echoes Goffman’s characterisation of the stigmatised person as rigid or impaired.

### Treating differently

According to Goffman, the separation induced by labelling with a stigmatising difference leads others to treat the stigmatised individual differently (Goffman, 1963). Differential treatment of the underperforming trainee thus implies that underperformance is stigmatising. One aspect of remediation that resulted in differential treatment was increased attention from the supervisor. Supervisors organised additional assessments, feedback from specialists, and meetings to discuss performance. For example, one supervisor said they were:

“More vigilant and more proactive in getting specific feedback, and yeah, I would increase my interactions and meetings with the individual” (#19).

Increased attention could also be more direct by manipulating rostering, with another supervisor saying they would “roster that trainee with the supervisors as much as possible… to increase our first-hand experience” (#14).

While this differential treatment appeared motivated by a desire to both help and further assess the trainee, by aligning with a marker of stigmatisation, it encourages the perception of underperformance as a stigmatising difference.

Modifying rosters and hence the trainee’s clinical exposure extended further when supervisors intervened to “take them off a particular [specialist] and pair them with someone different” (#17). Such manipulation of access to clinical opportunity could also extend to excluding the trainee from the after-hours roster. In one case, this was reported to disrupt the department and inconvenience other trainees, creating ill-feeling toward the trainee and further separation from their colleagues since the underlying reason was not disclosed to preserve trainee confidentiality. In these cases, the supervisor’s actions tangibly curtailed the trainee’s learning opportunities. While supervisors reported such decisions reflected their concern for patient and trainee safety, the effect resembles the decreased ‘life chances’ associated with stigma (Goffman, 1963, p5).

Beyond decreased access to clinical experience, trainees reportedly also feared longer term consequences that might be directly attributed to stigma:

“One of the things he was worried about was this was going to affect his Fellowship and work and potential employment at other hospitals.” (#16).

Such a comment acknowledges the trainee in question was concerned with the stigma of underperformance, lasting beyond the local circumstances. While supervisors appear not to intend to stigmatise trainees in their remediation practices, their actions do resemble the expected response to a stigmatising difference and thus may reinforce stigma.

## Discussion

This study explored how supervisors’ reported behaviour toward underperforming trainees in their assessment practices may reflect or induce stigma. We have used Goffman’s stigma theory to frame our theory-driven thematic analysis (Braun & Clarke, 2022; Goffman, 1963). Stigma is associated with underperformance (Chou et al., 2019; Ellaway, 2023). Our results build upon this general understanding by showing how our participants’ reported words and actions align with the processes of stigmatisation. Thus, in exploring supervisor experience in judging and remediating trainees, we have detailed the potential for stigma to influence practice and for practice to reinforce stigma.

We suggest perfectionism and sustained high achievement are expectations of trainees that become disrupted by underperformance; this aligns with the observation of mental health stigma in medical training (Bynum & Sukhera, 2021). Previous research has highlighted the incongruity between the demand for perfection in medical practice and our imperfect human nature (Sawatsky et al., 2023) and the stress that this places on trainee identity formation. Our results extend these findings by showing how this incongruity manifests when it shifts from the private to the public domain; formalising the discrepancy between the expectation of perfection and the reality of human frailty with an assessment decision and remedial process diminishes the trainee in the eyes of others. It is little wonder that no one wants to have these discussions or communicate these decisions.

Stigma may not motivate the stigmatising behaviours: for example, concealing may represent a genuine desire to protect privacy rather than seeing the trainee as somehow different. However, trainees entering remediation and the rest of their community of practice likely already recognise the social signals surrounding the stigmatising nature of underperformance (Goffman, 1963; Shaker et al., 2023). These unconscious learned attitudes and behaviours may undermine the supervisor’s virtuous attempts to avoid stigma (Phelan et al., 2014). Although motivated to minimise the trainee’s exposure to the effects of stigma, these efforts paradoxically emphasise the stigmatising nature of underperformance.

While the lens of stigma appears to reinforce the challenges of working with underperformance, Goffman provides us with some hope: ‘However, the perceived undesirability of a particular personal property, and its capacity to trigger off these stigma-normal processes, has a history of its own, a history that is regularly changed by purposeful social action.’ (Goffman, 1963, p138). Since stigma is a sociocultural phenomenon, it is within our collective capacity to change. The broader stigma literature recognises that interventions to address stigma must be multifaceted if they are to succeed (Link & Phelan, 2001). From our data, we have identified three potential targets for intervention:

1. Underlying beliefs and their cultural and structural supports.
2. Actions that mimic and hence induce stigma.
3. Addressing the label of ‘lacking insight’.

How might we address the underlying expectation that trainees need always perform well or achieve high scores? While it might be possible to meet high expectations in undergraduate education, postgraduate education is nested in the inherently more complex world of work. It is thus less amenable to simplistic scored assessments (Bynum & Sukhera, 2021). Helping trainees shift from a performance focus to more of a growth mindset (Dweck, 2012) with a mastery focus may provide one way to help trainees move away from a perfectionist mindset (Kalet & Zabar, 2023; Mills, 2023). The term remediation might usefully be replaced by development– a process that is relevant and necessary at all stages of expertise (albeit more supported in some phases).

Challenging the cultural expectation that trainees will progress through training in the minimum available time might also help destigmatise remediation. Introducing competency-based medical education, which aims to accommodate learners progressing at different rates and rely on assessment rather than time to determine progression (Lucey et al., 2018), might help. The training program studied already provides for time-variable training. Unfortunately, these rules have not changed the culture. Cultural change requires both individual and systemic effort to change practice (Bearman et al., 2021). Other programs implementing time-variable training would be wise to go beyond adopting new rules and address embedded cultural expectations.

Another targeted area for change could be reconsidering supervisor actions that mimic a stigmatising response. Supervisors avoided discussing underperformance, conducted remediation secretly and did not explain the reasons for differential treatment; these behaviours all signify that underperformance is a stigmatising difference, despite intentions. Supervisors who wish underperformance not to induce stigma may need to start acting as if they believe it is not stigmatising. Taking this a step further, Bynum and Sukhera (2021) refer to the notion of ‘stigma-sensitive care’ when discussing the stigma associated with mental illness. The approach they advocate for mental health may also improve trainee experience in remediation. To paraphrase, this would involve acknowledging the vulnerability required to accept and disclose underperformance, and helping trainees deal with internalised stigma and their fears of disclosure or remediation activities.

Labelling the trainee as lacking insight threatened the severest outcomes we would expect to see from stigma: discrimination; blaming; and exclusion. Insight has been described as an awareness of the difference between self-assessment and external assessment (Price et al., 2023). This definition does not capture the depth of disagreement a lack of insight implies in our study. Our supervisors wanted more than the trainee acknowledging a discrepancy between viewpoints; they wanted the trainee to accept the supervisor’s view of them and how they ought to behave in response. Insight, in our case, aligns with Goffman’s view that to accept the stigmatised person we expect them to conform to what we think (Goffman, 1963). The supervisors’ ability to expect and enforce this conformity and punish its absence draws our attention to their structural power and the hierarchical nature of medical training. Stigmatisation depends on power (Link & Phelan, 2001), and supervisors’ ability to label the trainee as lacking insight and subsequently stereotype them illustrates the role power plays.

Recognising that trainees may either hold an alternative view or, in fact, not wish to ‘confess’ the stigmatising difference (Bearman et al., 2018) may provide a different perspective. Supervisors might turn to remediation without a demand for insight by aiding the trainee’s own judgement (Price et al., 2021); more attention could be paid to pedagogical strategies to help trainees make meaning of their own performance (Bearman et al., 2023). Also, self-determination theory (Ryan & Deci, 2000), which focuses on competence, autonomy, and relatedness, can provide a basis for learner improvement practices that rely less on insight and supervisor-trainee agreement (Bearman et al., 2018). Although underperformance undermines trainees’ feelings of competence, supervisors could promote trainee autonomy by giving trainees greater responsibility for planning remediation and supplying evidence of improved performance (Bearman et al., 2018). Learner belonging might be enhanced by building a trusting relationship with a coach or mentor outside the assessment process (Price et al., 2021).

The challenge in addressing stigma is that it rests on the power of a dominant group to determine that a difference is stigmatising (Link & Phelan, 2001). Changing the beliefs and attitudes of supervisors and the broader community of practice using the approaches outlined offers hope that the stigma associated with underperformance can be reduced.

### Limitations and further research

Our study is situated in anaesthesia training in Australia and New Zealand and, while stigma in remediation is a collective problem (Regehr, 2010), readers will need to judge the applicability of our results to their context. Our interviews were performed with a different, although related, purpose, and thus, the questions asked did not directly address stigma, which might have limited the information available to us for analysis. However, it was stigmatising actions and behaviours evident in the data that piqued our interest and prompted this study. Moreover, we cannot make inferences about whether the stigma of underperformance *caused* supervisor practices, but we did infer that these practices *likely* had stigmatising effects.

Research examining the stigma of underperformance from the trainee point of view, supplementing the reports we have of supervisors’ views of stigma with first-person accounts, would complement this research. Emotions have been noted to play an important role in the trainee experience of remediation (Mills, 2023), which we could see some manifestation of in our data, but it would be interesting to investigate how this relates to stigma.

## Conclusion

Making assessment decisions and remediating trainees are fundamental requirements for any health professions training program. We found that stigmatising language and actions associated with underperformance can permeate these processes, impeding supervisors’ management and increasing trainees’ suffering. In particular, labelling a trainee as ‘lacking insight’ provided a stark example of stereotyping and its consequences. We suggest a multifaceted approach to modify practices and structures that reflect or reinforce stigma and address the underlying belief disrupted by underperformance. While such processes are both individually and collectively challenging, they lay out a path towards positive cultural change.

## Data Availability

No datasets were generated or analysed during the current study.
